# Comparing the Effect of Intralesional 5-Fluorouracil (5-FU) Alone Versus Intralesional 5-FU Combined With Triamcinolone Acetonide for Keloid Treatment

**DOI:** 10.7759/cureus.84635

**Published:** 2025-05-22

**Authors:** Sadaf Saleem, Mah e Neema Asghar, Abdullah Umer, Sofia Ahmed, Muhammad Umair Adeel, Humna Khan, Muhammad Hamza, Sumbal Jawad

**Affiliations:** 1 Dermatology, Sheikh Zayed Hospital, Lahore, PAK; 2 Dermatology, Bahawal Victoria Hospital, Bahawalpur, PAK; 3 Dermatology, Combined Military Hospital (CMH), Lahore, PAK; 4 Internal Medicine, Fatima Memorial Hospital, Lahore, PAK; 5 Otolaryngology, Combined Military Hospital (CMH), Lahore, PAK; 6 General Surgery, Muzaffarabad General Hospital, Muzaffarabad, PAK; 7 Dermatology, DHQ Teaching Hospital, Gujranwala, PAK

**Keywords:** 5-fluorouracil, intralesional, keloids, triamcinolone acetonide, vancouver scar scale

## Abstract

Introduction: Keloid is a benign hyper-proliferative growth of dermal fibroblasts, which extends beyond the borders of the original wound. It does not regress spontaneously and has the tendency to recur after excision.

Objectives: To compare the efficacy of intralesional 5-fluorouracil (5-FU) alone versus intralesional 5-FU combined with triamcinolone acetonide (TCA) in the treatment of keloids.

Methodology: This prospective observational study was conducted at the Department of Dermatology, Sheikh Zayed Hospital, Lahore, Pakistan, from July 2018 to July 2019. In this study, 66 cases, 33 in each group of aged 15-50 years having keloid of at least three or more on the Vancouver scar scale (VSS) score lasting less than five years, were included. The 66 patients were categorized into two equal groups. Group A was treated with 5-FU alone, and group B with 5-FU combined with TCA. Efficacy was defined as achieving a VSS score of 0 after eight weeks of treatment.

Results: From the 66 patients, there were 19 (57.58%) males in group A and 15 (46.88%) in group B. There were a total of nine (27.27%) cases of diabetes mellitus (DM) in group A and eight (24.24%) in group B. There were 12 (36.36%) and 10 (30.30%) hypertensive cases in groups A and B, respectively. Compliance was 100% in both groups. Efficacy in group A was seen in 25 (75.76%) cases and 32 (96.97%) cases in group B, with a p-value of 0.02. This efficacy was significantly better in group B in terms of male gender, where it was seen in 100% of cases in group B compared to 13 (68.42%) of cases in group A, with a p-value of 0.02. There was no significant difference in terms of age groups, BMI, and hypertension (HTN). The efficacy in diabetics was nearly significantly better in group B, where it was seen in 100% of the cases (p = 0.08).

Conclusion: Both groups are efficacious for the treatment of keloid; however, the combination of 5-FU and TCA revealed greater efficacy, and it was significantly better than 5-FU alone. The results were again significantly better in terms of efficacy in the male gender and those who had keloids for more than one year.

## Introduction

Keloids are the result of an overgrowth of dense fibrous tissue that usually develops after healing of a skin injury. The tissue extends beyond the borders of the original wound, does not usually regress spontaneously, and tends to recur after excision. The ancient Egyptians wrote about keloidal developments through their surgical procedures during the year 1700 BCE. In 1806, Alibert defined cheloide, which came from the Greek chele, meaning crab claw, to describe tissue growth into unaffected skin areas [1].

Different from keloids are hypertrophic scars (HTSs), which appear as pinkish red, raised, fibrous, inflamed areas that stay confined to the wounded area while they occasionally heal partly on their own. Necrotic tissue connects with the deep dermis when these types of injuries occur frequently [2]. The healing process of typical wounds gives rise to two distinct outcomes known as keloids and HTSs. The traditional wound healing process reaches balance when anabolic and catabolic activities meet about six to eight weeks after an injury takes place [3]. During this period, the wound has achieved strength equal to 30-40% compared to normal skin. Tensile strength of the scar improves during maturation because collagen fiber cross-linking occurs progressively. Wound healing leads to a scar becoming hyperemic and potentially thick, but this changes into a flat, mature, white, pliable scar, which might extend in size throughout numerous months [4]. During pathological wound healing, the production of collagen exceeds the breakdown rates, resulting in the growth of scars in multiple directions. The tissue stands as a raised scar that stays intensely red in color [5]. Medical experts divide excessive fibrous tissue into two categories: keloid and HTSs. The body develops keloids that expand beyond normal scar tissue boundaries after previous tissue trauma occurs. Keloids surpass the boundaries of trauma while protruding above surrounding skin levels, although they never penetrate beneath the subcutaneous tissue layer [6]. HTSs stick to the trauma site but self-regress between one and two years while they sometimes leave behind trace scarring. Keloids maintain different degrees of firmness, which may range from doughy and rubbery and even become completely rigid. Research shows proper methods to distinguish and categorize keloids by tactile examination. Early lesions are often erythematous. The aging process of HTSs leads to their transformation from brownish red to pale colored lesions [7]. These skin lesions contain no hair follicles, together with other functional adnexal glands. The rise of keloids that matches rising rates of skin pigmentation points to either a genetic origin or a minimal genetic connection between the two conditions [8].

Studies determine trauma to the skin from physical causes, such as earlobe piercing or surgery, and pathological causes such as acne or chickenpox, as the leading triggers for keloids [9]. From a physical and infection-related perspective, as well as from skin tension and abnormal matter's presence inside the body, different individuals form keloids or HTSs. The pathogenesis of the disease includes involvement of transforming growth factor-beta and adiponectin [10].

Objective 

To compare the efficacy of intralesional 5-fluorouracil (5-FU) alone versus intralesional 5-FU combined with triamcinolone acetonide (TCS) in the treatment of keloids.

The rationale for selecting these treatment modalities lies in their established pharmacological actions: 5-FU inhibits fibroblast proliferation and collagen production, while TCA reduces inflammation and suppresses abnormal collagen synthesis. By evaluating these treatments individually and in combination, this study aims to determine the most effective therapeutic approach for keloid regression.

## Materials and methods

Material and methods

This prospective observational study was conducted at the Department of Dermatology, Sheikh Zayed Hospital, Lahore, Pakistan, from July 2018 to July 2019. Data were collected through a non-probability consecutive sampling technique from 66 patients. The 66 patients were categorized into two equal groups, 33 in each group. Group A was treated with 5-FU only, and group B with 5-FU combined with TCA. This study focused primarily on clinical outcomes based on an objective Vancouver scar scale (VSS) scoring rather than patient-reported outcomes. The VSS is a validated clinical tool that assesses vascularity, pigmentation, pliability, and height of scars, with a score of 0 indicating complete resolution.

Sample size

The sample was calculated by selecting a statistical services calculator (internet) with 33 in each group by keeping the confidence equal to 95% and power equal to 80%, and prevalence of efficacy in cases of keloids with 5-FU alone as 72% and 5-FU with TCA as 96% in previous studies [5].

Inclusion criteria

The study included patients aged between 15 and 50 years, of either gender, presenting with a keloid having a VSS score of 3 or higher, with a duration of keloid of less than five years.

Exclusion criteria

The exclusion criteria included patients with a history of chronic renal failure or decompensated liver disease, as determined through medical history and records. Additionally, patients with a white cell count of less than 4,000/mm³, pregnant individuals (assessed by history and medical records), and those with known sensitivity to either of the drugs used in the study were excluded.

Data collection procedure

After the approval of this study from the College of Physicians and Surgeons, Pakistan, informed consent was obtained from all participants prior to their inclusion in the study. All participants underwent assessment for socio-demographic and clinical data. The data taken from all the subjects presenting to the Outpatient Department of Dermatology, Sheikh Zayed Hospital, Lahore, and recorded on a proforma include age, weight, height, BMI, diabetes mellitus (DM) (assessed by blood sugar reading (BSR) more than 140 mg/dL on two separate occasions 12 hours a part), hypertension (HTN) (assessed by systolic blood pressure (BP) of more than 140 mmHg on two separate occasions 12 hours a part), scores on the VSS duration of keloid (in months), and duration of symptoms (itching, discomfort). The cases in group A underwent treatment with 5-FU intralesionally in a strength of 50 mg/mL, while those in group B were treated with 0.7 mL of 5-FU in a strength of 50 mg/mL and 0.3 mL of TCA in a strength of 40 mg/mL combined in a single syringe. In both groups, 0.1 mL of the respective solution was injected in an area of 1 cm^2^ with the help of a 27-gauge insulin syringe, with aseptic measures, by a medical officer with at least one year of experience in dermatology. The treatment was given weekly at the Dermatology OPD for a total of eight weeks. After treatment, they were followed weekly for another eight weeks to look for the VSS score for keloid by a medical officer with at least one year of experience in dermatology under the supervision of a supervisor. Efficacy was defined as achieving a VSS score of 0 after eight weeks of treatment. The medical personnel recorded the results using an identical proforma.

Data analysis

Data were analyzed with the help of Statistical Product and Service Solutions (SPSS, version 19; IBM SPSS Statistics for Windows, Armonk, NY). Quantitative variables, such as age, weight, height, BMI, pre-treatment and post-treatment VSS scores, duration of keloid, and duration of symptoms, were presented in terms of mean ± standard deviation (SD). Frequency and percentages were calculated for gender, DM, HTN, compliance (which was labeled yes when the patient visited every time and underwent treatment assessed by the on-duty doctor examining the keloids), and outcome (i.e., efficacy: yes or no). Time taken to efficacy was noted. Two groups were compared for efficacy by applying the chi-square test, and p-value ≤0.05 was considered significant. Effect modifiers were controlled through stratification of age, gender, BMI, DM, HTN, duration of keloid and its symptoms, and compliance with treatment, to see their effect on the outcome variable. The post-stratification chi-square test was also applied to look for the effect of effect modifiers on outcome, and p ≤0.05 was considered significant.

## Results

The analysis of the two groups, A (n = 33) and B (n = 33), revealed no significant differences in age (p = 0.92), weight (p = 0.69), or height (p = 0.09). However, a significant difference was observed in BMI, with group B showing a higher mean BMI (30.06 ± 3.96) compared to group A (27.91 ± 4.43), with a p-value of 0.01. The range for all variables was similar between the two groups, with both having participants spanning from 15 to 50 years of age, and similar extremes in weight, height, and BMI (Table 1).

**Table 1:** Demographics of patients in group A and group B BMI: Body Mass Index, SD: Standard Deviation, CI: Confidence Interval

Variable	Group A (n = 33)	Group B (n = 33)	p-value
Age (years)	Mean ± SD: 33.18 ± 12.13	Mean ± SD: 33.27 ± 11.74	0.92
	Median: 30.00	Median: 34.00	
	95% CI: 28.88–37.48	95% CI: 29.11–37.44	
	Minimum: 15, Maximum: 50	Minimum: 16, Maximum: 50	
Weight (kg)	Mean ± SD: 63.12 ± 19.70	Mean ± SD: 64.15 ± 17.69	0.69
	Median: 59.0	Median: 65.0	
	95% CI: 56.13–70.11	95% CI: 57.88–70.42	
	Minimum: 24, Maximum: 98	Minimum: 25, Maximum: 99	
Height (cm)	Mean ± SD: 154.21 ± 13.88	Mean ± SD: 158.12 ± 13.72	0.09
	Median: 156.00	Median: 157.00	
	95% CI: 149.29–159.14	95% CI: 153.26–162.99	
	Minimum: 129, Maximum: 180	Minimum: 129, Maximum: 189	
BMI (kg/m²)	Mean ± SD: 27.91 ± 4.43	Mean ± SD: 30.06 ± 3.96	0.01
	Median: 28.00	Median: 29.00	
	95% CI: 26.34–29.48	95% CI: 28.66–31.46	
	Minimum: 21, Maximum: 35	Minimum: 23, Maximum: 39	

The comparison between group A (n = 33) and group B (n = 33) showed no significant difference in the duration of keloid (p = 0.13) or the pre-treatment VSS score (p = 1.00). However, significant differences were found in post-treatment VSS scores (p = 0.04) and time to efficacy (p = 0.03). Group B showed a lower post-treatment VSS score (0.06 ± 0.34) compared to group A (0.48 ± 0.93) and a shorter time to efficacy (4.85 ± 0.61 weeks vs. 5.94 ± 0.93 weeks) (Table 2).

**Table 2:** Study variables in group A and group B VSS: Vancouver Scar Scale, SD: Standard Deviation, CI: Confidence Interval

Variable	Group A (n = 33)	Group B (n = 33)	p-value
Duration of keloid (yrs)	Mean ± SD: 2.03 ± 0.95	Mean ± SD: 1.70 ± 0.46	0.13
	Median: 2.00	Median: 2.00	
	95% CI: 1.69–2.37	95% CI: 1.53–1.86	
	Minimum: 1, Maximum: 4	Minimum: 1, Maximum: 2	
Pre-treatment VSS score	Mean ± SD: 3.85 ± 0.75	Mean ± SD: 3.85 ± 1.00	1.00
	Median: 4.00	Median: 4.00	
	95% CI: 3.58–4.12	95% CI: 3.49–4.20	
	Minimum: 3, Maximum: 5	Minimum: 3, Maximum: 7	
Post-treatment VSS score	Mean ± SD: 0.48 ± 0.93	Mean ± SD: 0.06 ± 0.34	0.04
	Median: 0.00	Median: 0.00	
	95% CI: 0.15–0.82	95% CI: -0.06 to 0.06	
	Minimum: 0, Maximum: 3	Minimum: 0, Maximum: 2	
Time to efficacy (weeks)	Mean ± SD: 5.94 ± 0.93	Mean ± SD: 4.85 ± 0.61	0.03
	Median: 6.00	Median: 5.00	
	95% CI: 5.61–6.27	95% CI: 4.63–5.07	
	Minimum: 4, Maximum: 8	Minimum: 4, Maximum: 6	

In terms of efficacy, group B showed a significantly higher success rate compared to group A. Specifically, 96.97% (32/33) of participants in group B experienced efficacy, while 75.76% (25/33) in group A did. This resulted in a total of 86.36% (57/66) efficacy across both groups, with only 13.64% (9/66) of participants not achieving efficacy (Table 3).

**Table 3:** Comparison between two groups in terms of efficacy

Group	Efficacy		Total
	Yes	No	
A	25 (75.76%)	08 (24.24%)	33 (100%)
B	32 (96.97%)	01 (03.03%)	33 (100%)
Total	57 (86.36%)	09 (13.64%)	66 (100%)

For male participants, group B demonstrated a significantly higher efficacy rate (100%) compared to group A (68.42%), with a p-value of 0.02. In contrast, for female participants, there was no significant difference in efficacy between the two groups (p = 0.57), with group A showing 85.71% efficacy and group B showing 94.12% efficacy. Overall, male participants showed a total efficacy of 82.35%, while female participants had a slightly higher total efficacy of 90.32% (Table 4).

**Table 4:** Efficacy in both groups with respect to gender

Gender		Efficacy		Total	Test Statistic (p-value)
		Yes	No		
Male	Group A	13 (68.42%)	06 (31.58%)	19 (100%)	χ²(1) = 5.44, *p* = 0.02 Cramer’s *V* = 0.40
	Group B	15 (100%)	00 (00%)	15 (100%)	
	Total	28 (82.35%)	06 (17.65%)	34 (100%)	
Female	Group A	12 (85.71%)	02 (14.29%)	14 (100%)	χ²(1) = 0.32, *p* = 0.57 Cramer’s *V* = 0.10
	Group B	16 (94.12%)	01 (53.5%)	17 (100%)	
	Total	28 (90.32%)	03 (09.68%)	31 (100%)	

For the age group 15-32 years, group B had 100% efficacy, while group A showed 83.33%, but the difference was not statistically significant (p = 0.35). For the 33-50 years age group, group B again showed a higher efficacy (95.45%) compared to group A (74.07%), though this difference also was not statistically significant (p = 0.06). Overall, efficacy was higher in group B across both age groups, but the differences in efficacy between groups within these age ranges were not significant (Table 5).

**Table 5:** Efficacy in both groups with respect to age group

Age Group (Years)		Efficacy		Total	Test Statistic (p-value)
		Yes	No		
15-32	Group A	05 (83.33%)	01 (16.67%)	06 (100%)	χ²(1) = 1.06, p = 0.35 Cramer’s *V* = 0.25
	Group B	11 (100%)	00 (00%)	11 (100%)	
	Total	16 (94.12%)	01 (05.88%)	17 (100%)	
33-50	Group A	20 (74.07%)	07 (25.93%)	27 (100%)	χ²(1) = 3.51, p = 0.06 Cramer’s *V* = 0.27
	Group B	21 (95.45%)	01 (04.55%)	22 (100%)	
	Total	41 (83.67%)	08 (16.33%)	49 (100%)	

In the group with a keloid duration of one year or less, there was no significant difference in efficacy between group A (90%) and group B (100%), with a p-value of 1.0. However, in the group with a keloid duration of more than one year, group B showed significantly higher efficacy (95.65%) compared to group A (69.56%), with a p-value of 0.04 (Table 6).

**Table 6:** Efficacy in both groups with respect to the duration of keloid

Duration of Keloid	Group	Efficacy: Yes	Efficacy: No	Total	Test Statistic (p-value)
1 year or less	Group A	09 (90%)	01 (10%)	10 (100%)	χ²(1) = 0.00, p = 1.00 Cramer’s *V* = 0.00
	Group B	10 (100%)	00 (00%)	10 (100%)	
	Total	19 (95%)	01 (5%)	20 (100%)	
> 1 year	Group A	16 (69.56%)	07 (30.44%)	23 (100%)	χ²(1) = 4.21, p = 0.04 Cramer’s *V* = 0.30
	Group B	22 (95.65%)	01 (4.35%)	23 (100%)	
	Total	38 (82.60%)	08 (17.4%)	46 (100%)	

## Discussion

HTSs and keloid scars are common dermatological complaints produced by disruption of the normal wound-healing process. Despite a wide array of therapeutic options available to treat these lesions, HTSs and keloids continue to pose a significant challenge to clinicians in everyday practice. The chemotherapeutic drug 5-FU is a well-known treatment option reserved for recalcitrant HTSs and keloid lesions [11]. The current evidence suggests that 5-FU is a safe and practical alternative for the treatment of HTSs and keloids as it may substantially improve the appearance of proliferative scars and reduce the chance of recurrence. This therapeutic option is most effective in conjunction with adjuvant therapy, such as corticosteroids [12]. However, additional randomized controlled clinical trials with large sample sizes should be conducted to corroborate the existing efficacy and safety data in patients with HTSs and keloids. Efficacy in group A was seen in 25 (75.76%) and 32 (96.97%) of cases in group B, with p values of 0.02. According to a study by Sharma et al., good-to-excellent response was seen in 72% of cases that were treated with 5-FU only, and 96% got relief in the group with 5-FU and TCA given together [13,14]. In another study by Khan et al., the from Pakistan, 5-FU and TCA combination was found statistically significantly better combination, and it revealed good-to-excellent results in 68% of cases with 5-FU and 84% with 5-FU and TCA [15]. In a study by Darougheh et al., it was seen that a good response was seen only in 15% with 5-FU and 40% with both 5-FU and TCA [16,17].

According to a study by Davison et al., a combination of 5-FU and triamcinolone intralesional steroid therapy is better than the steroid only in the treatment of keloids [18]. The patients who received 5-FU/steroid combination had a 92% average reduction in the size of lesions compared to 73% in the group of patients who received steroid alone. The results were found to be statistically significant (p = 0.05) [19]. According to a study by Wu et al., this combination of drugs was offered after surgical resection of keloid, and it was considered an effective method to treat auricular keloid and prevent its recurrence [20]. Normal auricular shapes were achieved in 77 (92.7%) cases, and the total effective rate was 100% [21]. The reason that intralesional TCA alone is less effective than combination therapy with intralesional TCA and 5-FU is that, in addition to inhibitory effect on TGF-ß-induced expression of type 1 collagen gene in human fibroblasts, 5-FU has an inhibitory effect on production of thymidylate synthetase, thus blocking DNA synthesis. By inhibition of DNA synthesis in rapidly proliferating and metabolizing cells, apoptosis of fibroblasts is increased [22]. In comparison with the TCA group, it seems that TCA+5-FU combination is more effective and provides a more rapid response with fewer side effects. Apikian et al. found that intralesional 5-FU mixed with low-dose corticosteroid may be a possible alternative for keloid scar [23-26].

In the present study, two significantly better parameters were seen. One of them was the male gender, and the other one was the duration of the keloid for more than one year. This efficacy in group B in terms of male gender was seen in 100% of cases in group B as compared to 13 (68.42%) of cases in group A (p = 0.02). Moreover, the cases that had keloid for more than one year showed a response to group B therapy, where it was seen in 22 (95.65%) as compared to 16 (69.56%) with group A (p = 0.04). There was not much work done regarding these cutoff values in the studies conducted in the past. They studied the overall effects of all these agents, but one factor was common that the combination of 5-FU and TCA was significantly better than 5-FU alone. The reason for higher success in males as compared to females can be better tolerance and regular use of the medication. One of the common side effects observed in the present and past studies was pain at the local site and pruritus. The reason for better efficacy in cases where the duration of the keloid was more than one year can be explained by the fact that the longer the disease duration, the higher the chances of sticking to treatments, and these cases would have taken some other treatment in the past as well for these lesions.

Despite the valuable insights provided by this study, several limitations should be acknowledged. The relatively small sample size may limit the generalizability of the findings across broader patient populations. Additionally, while the study employed the VSS as an objective measure of treatment efficacy, it did not incorporate patient-reported outcomes or histological analyses, which could offer a more comprehensive assessment of treatment success, including patient satisfaction and quality of life. The follow-up period of eight weeks was relatively short, restricting the evaluation of long-term treatment effects and recurrence rates. Furthermore, potential confounding factors such as variations in skin type, previous treatments, and genetic predispositions were not extensively examined, which may influence response to therapy. Finally, although a detailed methodology was provided, further elaboration on injection techniques and monitoring for adverse effects could enhance reproducibility in future studies. Addressing these limitations in subsequent research will be essential to validate and expand upon the findings presented here.

## Conclusions

Both groups are efficacious for the treatment of keloids; however, the combination of 5-FU and TCA revealed greater efficacy, and it was significantly better than 5-FU alone. The result was again significantly better in terms of efficacy in the male gender and those who had keloids for more than one year.
